# Evolution in Dentistry

**Published:** 1890-08

**Authors:** J. A. Robinson


### Evolution in Dentistry.

BY J. A. ROBINSON, D.D.S.

Every thing is perfected by a gradual and natural process. All organic and inorganic forms if we could see from the beginning were in the mind of the creator before they were made or they would not have been, and the great end is *use.* It is the *end*, the *cause*, and the *effect*, and the end sought is before the cause and grows out of the motive that prompts to action and that is love, and this love or desire follows along in the path and in company with wisdom, until the thing is accomplished or completed. And this is evolution and it is always onward and upward. It is the ideal in whatever we desire to make perfect, and the ideal man is always greater than the object he wishes to attain.

The first thought in filling teeth was, and is, to preserve them for *use*, to make them useful. The next is ornamental, which is nearly the same thing. Soft gold foil being the most indestructible, suggested itself as the most useful and ornamental. The next was tin foil as it had more qualities corresponding to gold than any other metal. It is non-elastic, very ductile and when pushed into a cavity the crystals break, and in a bar make what is called the cry of tin. When combined with gold or platinnm it becomes rigid and elastic, and so to restore the non-elastic quality the writer made it fibrous in the manufacture of the fibrous and textile metallic filling that he introduced to the profession in 1878 for filling teeth and lining rubber dental plates.

The end to be attained being, use first, and ornament next, has led many dentists of the day to run into excess by making *show fillings* and sacrificing good healthy tissue to give place to a patch of gold for ornamentation. It is an infinitesimal relic of barbarism in man, as we learn that among the barbarous and savage nations ornamentation oftentimes preceded the dress of the savage tribes in the remote corners of the earth. The ideal dentist of fifty years ago was in most cases a doctor of medicine as well as a dentist and his ambition was only to save the teeth; his only thought was how best to preserve them and prevent the gold exposure; consequently contour fillings were unknown. In carving block work for a dentist in Boston, in 1850, I made some cavities in a front tooth for a Dr. Clough, whose office was in Tremont Temple, (at his suggestion) to match a natural front tooth that had been filled, and that was the first time I had ever known an artificial tooth to be filled. I manufactured all the teeth I used for more than twenty-five years. There were some advantages in filling teeth with soft foil and by hand pressure. First, there was no temptation to make show fillings, and if the dentist had a good hand and suitable instruments there was no danger of cracking the enamel if he filled with soft foil, and then he packed all the gold around the borders of the cavities first, and could repeat the process and avoid pressure over the pulp cavity and prevent pain after filling. I always fill the borders of the cavity to-day with soft foil and finish with cohesive foil. The finish of cohesive foil is the improved method of the old plan and is evolution. The dental profession started with the doctor of medicine but differentiated by a large influx of persons with an adventuous spirit and large mechanical skill of the finer sort who discarded conventionalities and codes, and thus swept past the average doctor of medicine in dental science and literature who had only paid attention to diseases incident to carelessness, climate, or accident in place of his home and surroundings. So the dentist paid no attention to the diseases that belong directly to the doctor of medicine until dental colleges and education brought them nearer together.

The practice of dentistry is more thoroughly allied to art than the medical doctor, for he converts values into *uses*. We do not create *values*, they are the gift of the Creator. We only create *uses of values* as they are bestowed in nature, and whenever we discover our mistakes and discontinue any *established use* that is injurious to humanity we make progress in science, in art, or religion, and that is evolution. To discontinue the practice of extracting every aching tooth and curing pyorrhœa alveolaris, regulating teeth by modern appliances and the gentle process is evolution in practical dentistry. Capping exposed pulps whereby ninety-five per cent. of the aching teeth can be saved without the use of arsenious acid is evolution. Learning that it is inadmissible to use any instrument when the pulp is exposed but a spoon-shaped excavator to remove the coriaceous or leathery concretions that nature has deposited to save the life of the tooth is evolution in practice.

To remember that in excavating a cavity the instrument should cut toward the cutting edge of the tooth is evolution in practice. In the use of arsenic to devitalize the pulp to apply the infinitesimal dose with carbolic acid and then remove the pulp with the Donaldson barbed nerve needle wet with carbolic acid in six hours with gentleness and great care, and it can be done absolutely without pain. The pain is not caused by removing the pulp, but by the air that presses upon the transverse nerves through the foramen, and it is another evolution in dental practice.

Some seven years ago I wrote an article on the use of carbolic acid and caustic potash as a pyorrhœa cure, and obtunder for sensitive dentine. My hypothesis was, that in a sensitive tooth it coagulated the serum in the tubuli and cut off all connection with the pulp so the operation would be painless. My predictions in regard to its merit have been more than realized. The dental depots of the country have made an imperfect article and called it "Robinson's Remedy" it is a poor imitation of the genuine remedy that has led some to doubt the efficacy of the cure. I have received hundreds of letters commending it, and sold hundreds of bottles all over the country to our best operators who use it constantly. It is ten times as effective as cocaine, and a hundred times less injurious than arsenic. I believe it to be a retrograde movement to use the dental engine after the pulp cavity has been thoroughly cleansed, especially in teeth back of the cuspids, for the nerve canals are uncertain in their direction, and injury to the cementum is disastrous, though the alveolar process will, from its honey-comb nature, bear almost any amount of malpractice and abuse. Chloroform is king of pain when there is any inflammation of the alveolar process after a dental operation. It reaches up under the free margin of the gum to the cementum, through the honey-comb alveolar process, and is an anæsthetic as much as carbolic acid which is an alcoholic intoxicant and makes *dead drunk*, or paralyzes the pulp of the tooth till it can be destroyed and removed. It is a sort of *twilight* vision of perfect dentistry that we have to-day. Paul in his vision of the glories of the Resurrection says, "It doth not yet appear what we shall be."

Some of the rainy days of the present month when it was cold and wet I kept singing to "May:"

O! gentle, loving May
Why do you thus delay
Your coming and your bringing of the flowers?
Why make our hearts so sad
When we should be so glad
To see the buds unfolding by your powers?

Come and put forth the leaves
On all the forest trees.
So that the birds may shelter well their nests;
They can not bear to wait
Since they have found a mate,
And need a place their loving hearts to rest.

Destroy the snowy shrouds
That hide the soft blue clouds,
And send a chill to every human heart.
Come with your bright, warm sun
And say that spring's begun,
And summer will be here e'er she depart.

Come cloth the grass with green,
Come with your bluest sheen,
And fatten all the cattle on the hills.
The maples look so sad,
The squirrels are afraid
To bring their young to play among the rills.

The hope of all the race
Is in thy promised face ;
The plowboy now his furrow has unfurled;
Send forth thy lightning strong,
Thy thunders loud and long,
Thy fireworks blazing from a distant world.

Then will the summer come,
And autumn follow on
With all the fulness of her bounteous store,
And each succeeding year
Will make the fruits appear,
Till earth, and heaven, and time shall be no more.

